# The MASLD Journey in the General Population: Linkage‐to‐Care and Patient‐Reported Uptake of Fibrosis Risk Assessment

**DOI:** 10.1111/liv.70658

**Published:** 2026-04-20

**Authors:** Joo Hyun Oh, Jun‐Hyuk Lee, Sang Bong Ahn, Eunjoo Kwon, Eileen L. Yoon, Hyo Young Lee, Seon Cho, Dae Won Jun

**Affiliations:** ^1^ Department of Gastroenterology CHA University School of Medicine Seongnam Republic of Korea; ^2^ Department of Family Medicine, Nowon Eulji Medical Center Eulji University College of Medicine Seoul Republic of Korea; ^3^ Department of Internal Medicine, Nowon Eulji Medical Center Eulji University College of Medicine Seoul Republic of Korea; ^4^ Medicheck Research Institute Korea Association of Health Promotion Seoul Republic of Korea; ^5^ Department of Internal Medicine Hanyang University, College of Medicine Seoul Republic of Korea; ^6^ Hanyang Institute of Bioscience and Biotechnology Hanyang University Seoul Korea

**Keywords:** guideline implementation, linkage‐to‐care, MASLD

## Abstract

**Background & Aims:**

Despite the growing burden of steatotic liver disease (SLD), real‐world evidence on patient‐reported post‐diagnostic care pathways and fibrosis risk assessment remains limited. We aimed to describe the patient‐reported care pathway from initial diagnosis to subsequent follow‐up and fibrosis assessment among individuals with self‐reported SLD, including those meeting at‐risk criteria in a population‐based survey sample.

**Methods:**

A web‐based survey was conducted among Korean adults aged ≥ 19 years recruited from an established online research panel, using quota strata to approximate the national distribution of key demographics. Respondents completed a screening questionnaire; those who screened positive for self‐reported physician‐diagnosed or imaging‐detected fatty liver/SLD proceeded to the full survey on post‐diagnosis healthcare utilization and follow‐up. Of 12 946 respondents with a valid screening questionnaire, 1000 individuals with self‐reported SLD comprised the final analytic sample.

**Results:**

The self‐reported prevalence of physician‐diagnosed or imaging‐detected SLD was 23.7%. Most participants (79.9%) reported an incidental diagnosis during routine medical check‐ups. Overall, 57.7% (577/1000) reported linkage‐to‐care, and 14.9% (86/577) of those reporting linkage‐to‐care also reported having undergone fibrosis assessment. Among those reporting no follow‐up, the most commonly cited reason was the belief that SLD was not a serious condition. Among those reporting linkage‐to‐care, 68.8% (397/577) reported follow‐up in primary clinics and 31.2% (180/577) in referral centres; patient‐reported fibrosis assessment was 10.6% (42/397) and 24.4% (44/180), respectively. The at‐risk subgroup comprised 61.9% (619/1000); 65.6% (406/619) reported linkage‐to‐care and 12.1% (75/619) reported fibrosis assessment. Patient‐reported linkage‐to‐care was 55.5% in MASLD, 59.7% in MetALD and 65.2% in ALD.

**Conclusion:**

In this survey sample, most respondents with self‐reported SLD reported an incidental diagnosis during medical check‐ups, and patient‐reported fibrosis assessment was uncommon, including among those meeting at‐risk criteria.

## Research in Context

**Evidence Before This Study:**

- Steatotic liver disease (SLD) is common, but real‐world data are limited on what happens after diagnosis—especially whether people are linked to care and whether guideline‐recommended fibrosis assessment is performed, particularly in primary care and in at‐risk groups.

**Added Value of This Study:**

- In a web‐based, quota‐stratified survey sample of Korean adults, 1000 respondents with self‐reported physician‐diagnosed or imaging‐detected fatty liver/SLD were identified from 12 946 screened individuals. Most respondents reported an incidental diagnosis during routine medical check‐ups (79.9%).
- Only 57.7% (577/1000) reported linkage‐to‐care. Among those reporting linkage‐to‐care, 68.8% reported follow‐up in primary clinics and 31.2% in referral centres. Patient‐reported fibrosis assessment was uncommon (14.9% [86/577] overall) and was reported less often in primary clinics (10.6% [42/397]) than in referral centres (24.4% [44/180]).
- The at‐risk subgroup comprised 61.9% (619/1000). While linkage‐to‐care was higher in this subgroup (65.6% [406/619]), patient‐reported fibrosis assessment remained uncommon (12.1% [75/619]).

**Implications of All the Available Evidence:**

- Across the care pathway, substantial attrition may occur: many respondents reported no follow‐up after diagnosis, and reported uptake of fibrosis risk assessment was low even among those who re‐engaged with care and among those meeting at‐risk criteria. These findings suggest an important opportunity to strengthen post‐diagnostic follow‐up and to support broader, more consistent use of fibrosis risk assessment tools in routine care, particularly in primary care settings.

**Abbreviations**
ALD: alcoholic liver disease
AUDIT‐C: Alcohol Use Disorders Identification Test–Consumption
BMI: body mass index
CI: confidence interval
ELF: enhanced liver fibrosis
FIB‐4: fibrosis‐4 index
MASLD: metabolic dysfunction‐associated steatotic liver disease
NAFLD: non‐alcoholic fatty liver disease
SLD: steatotic liver disease
T2DM: type 2 diabetes mellitus

**Lay Summary**

In this patient‐reported survey of Korean adults, most individuals with metabolic dysfunction‐associated steatotic liver disease were incidentally diagnosed during routine health check‐ups. However, many reported no subsequent follow‐up, and uptake of fibrosis assessment was low even among high‐risk individuals. These findings highlight a substantial gap between diagnosis and guideline‐recommended fibrosis evaluation, underscoring the need to strengthen post‐diagnostic care pathways, particularly in primary care settings.

## 1 Introduction

Metabolic dysfunction‐associated steatotic liver disease (MASLD) has become the leading cause of chronic liver disease worldwide, with an estimated prevalence of approximately 25%–35% [1]. Although several novel pharmacologic therapies for MASLD have recently entered clinical practice [2, 3], accumulating evidence suggests that a substantial proportion of patients who would benefit from treatment and structured follow‐up do not receive appropriate evaluation or care [4, 5, 6].

In an effort to bridge this gap, many countries have proposed clinical pathways and practice guidelines aimed at identifying high‐risk MASLD, enabling timely fibrosis assessment and early intervention [7, 8, 9]. However, the real‐world dissemination of these pathways remains limited, particularly in community‐based healthcare systems. Prior studies have largely focused on awareness of MASLD in the general population and among healthcare professionals [10, 11, 12], whereas much less is known about what happens after steatotic liver disease (SLD) is identified in routine practice. In particular, it remains important to understand where patients with SLD are first diagnosed and whether that diagnosis leads to appropriate referral, linkage‐to‐care and subsequent clinical evaluation. Current guidelines place primary care at the centre of the initial identification of SLD and subsequent risk stratification [7, 13, 14, 15], yet the extent to which this pathway is reflected in routine care remains insufficiently characterized.

To improve linkage‐to‐care among individuals with SLD and to strengthen the implementation of guideline‐recommended risk stratification for high‐risk patients in primary care clinics, it is essential to characterize the real‐world care journey in the general population—including awareness of SLD, pathways to diagnosis and subsequent management after diagnosis.

Therefore, in this study, we conducted a structured nationwide web‐based survey of adults with self‐reported SLD to investigate, first, the patterns of diagnosis and subsequent linkage‐to‐care, and, in particular, the extent to which SLD with at‐risk population receive guideline‐recommended liver fibrosis assessment, and second, to characterize the patient‐reported uptake of guideline‐recommended fibrosis risk assessment in primary care clinics.

## 2 Method

### 2.1 Study Design

We conducted a nationwide, cross‐sectional survey to assess healthcare utilization and care pathways following a diagnosis of SLD among adults in Korea. A structured, web‐based self‐administered questionnaire was implemented by MACROMILL EMBRAIN Co. Ltd. (https://embrain.com/eng/).

### 2.2 Ethics

The study was conducted in accordance with applicable ethical standards and regulatory guidelines and was approved by the Institutional Review Board of Hanyang University (IRB‐2025‐03‐010). The IRB waived the requirement for informed consent because we used de‐identified data provided by the investigating company.

### 2.3 Survey Population and Sampling

Eligible participants were Korean adults aged ≥ 19 years who were registered members of an established online research panel. Participants were recruited from an established online research panel maintained by MACROMILL EMBRAIN Co. Ltd., which includes adults who voluntarily registered to participate in online surveys. Survey invitations were distributed randomly to panel members. The survey was conducted between August 25 and September 1, 2025. The MACROMILL EMBRAIN panel comprises over 1.5 million registered adult members with nationwide coverage and has been widely used for population health surveys in Korea (https://embrain.com/eng/company/embrain).

### 2.4 Survey Flow and Inclusion Criteria

Figure S1 summarizes the survey flow, eligibility screening and analytic sample derivation. To reduce potential framing effects, the specific study hypothesis was not disclosed at the initial contact. Instead, respondents were presented with a list of approximately 20 chronic conditions written in lay terms and asked to select all conditions they had been told they had or had been diagnosed with by a healthcare professional (multiple selections allowed). For SLD, the screening item was phrased as ‘fatty liver (hepatic steatosis)’, and respondents were instructed to select it only if they had (i) received a physician diagnosis of fatty liver/SLD or (ii) been informed of hepatic steatosis detected on imaging (e.g., abdominal ultrasonography) during a medical check‐up or clinical visit. This condition‐list approach—embedding the SLD item among other chronic conditions without disclosing the study focus—was designed to minimize social desirability and framing biases. In Korea, most SLD diagnoses originate from national health screening programmes that routinely include abdominal ultrasonography, and a written result letter specifying the diagnosis is provided to each examinee. This practice may facilitate accurate recall of a prior SLD diagnosis. The complete questionnaire, including the exact wording and response options for all screening items, is provided in Table S1 (items SQ4, Q1 and Q2). During the survey period, a total of 13 013 individuals accessed the survey link. Invitations were distributed to online panel members using a quota‐based sampling design intended to align the final sample with the Korean population distribution by age, sex, region of residence and household income. Of these, 67 individuals discontinued before completing the screening items, resulting in 12 946 respondents with a valid screening questionnaire. From this screening base, 3064 individuals (23.7%) screened positive for self‐reported fatty liver/SLD and were eligible for the full survey on post‐diagnosis healthcare utilization and management. However, 2014 eligible respondents were not invited to proceed to the full survey because the pre‐specified quota targets (by age, sex, region of residence and household income) had already been filled. Consequently, 1050 eligible respondents proceeded to and submitted the full survey. Following predefined data quality checks, 50 responses were excluded (e.g., implausibly short completion time or inconsistent/insincere responses to open‐ended items). Ultimately, 1000 respondents were included in the final analysis.

### 2.5 Questionnaire

A structured questionnaire was developed through multidisciplinary collaboration involving four hepatologists, a primary care physician in family medicine, nursing professionals from health check‐up centres and experts from a professional survey research organization. Guided by previous survey studies [10], the instrument was organized into six domains to capture key aspects of healthcare engagement among individuals with SLD. Baseline health status addressed underlying medical conditions and previous episodes of abnormal liver enzyme levels. Alcohol consumption was assessed using self‐reported measures, including the number of standard drinks consumed during the preceding week, average weekly alcohol intake and the Alcohol Use Disorders Identification Test–Consumption (AUDIT‐C). Reported alcohol intake was converted to grams of alcohol per day, assuming 10 g of alcohol per standard drink [16]. Diagnostic experience and healthcare utilization captured the setting of initial diagnosis—medical check‐up centres, primary clinic or general hospital—and actions taken afterward, such as no action, follow‐up at a primary care clinic or referral to higher‐level care. Fibrosis assessment was defined as patient‐reported receipt of non‐invasive fibrosis evaluation using imaging‐based elastography or blood‐based fibrosis tests. Specifically, fibrosis assessment was captured using multiple questionnaire items. Among respondents who reported linkage‐to‐care, item Q7 asked about tests or treatments received after SLD diagnosis, including a response option describing a liver fibrosis test assessing liver ‘stiffness’. In addition, item Q8 assessed awareness of fibrosis testing, and respondents who answered affirmatively were subsequently asked (Q9) whether they had undergone such testing. Because this measure relies on patient recall and recognition, serum‐based fibrosis indices (e.g., FIB‐4) calculated from routine laboratory tests may not have been captured if patients were unaware that such assessments had been performed. The full questionnaire is available in Table S1.

### 2.6 Definition of SLD Subtypes and At‐Risk Population

In this study, we used the term ‘SLD’ to reflect patient‐reported, whereas ‘MASLD’ was used when referring to the contemporary disease definition and guideline framework.

SLD subtypes were classified according to alcohol consumption patterns and metabolic dysfunction using a stepwise algorithm aligned with contemporary MASLD criteria [9]. MASLD, metabolic dysfunction and alcohol‐related liver disease (MetALD), and alcoholic liver disease (ALD) categories were determined based on the estimated average daily alcohol intake; participants were categorized as having low alcohol consumption (< 20 g/day for women or < 30 g/day for men), moderate‐to‐high consumption (20–49 g/day for women or 30–59 g/day for men) or high consumption (≥ 50 g/day for women or ≥ 60 g/day for men). Participants who self‐reported hepatitis B or C virus carriage were classified as Others. The SLD with ‘at‐risk population’ was defined as individuals with SLD who met at least one of the following criteria: (i) presence of type 2 diabetes mellitus (T2DM); (ii) obesity, defined as self‐reported body mass index (BMI) ≥ 25 kg/m^2^, accompanied by at least one additional cardiometabolic risk factor adopted from the MASLD definition (including hypertension, dyslipidemia, cardiovascular disease or chronic kidney disease); or (iii) persistently elevated liver enzymes, defined as alanine aminotransferase (ALT) or aspartate aminotransferase (AST) > 40 U/L on two or more occasions within the past 2 years [8].

### 2.7 Outcomes

The primary outcome was the linkage‐to‐care rate among individuals with SLD and those in the SLD at‐risk population. Linkage‐to‐care was defined as engagement in liver‐related follow‐up evaluation or clinical care within the healthcare system, either (1) being diagnosed with SLD during a visit for liver‐related concerns in a primary care clinic or secondary/tertiary hospital setting or (2) after an incidental SLD diagnosis made during care for non–liver‐related conditions, subsequently attending any clinic or hospital specifically for liver evaluation. The secondary outcome was patient‐reported uptake of non‐invasive fibrosis risk assessment using imaging‐based or blood‐based tests.

### 2.8 Statistical Analysis

Continuous variables were summarized as means with standard deviations, and categorical variables as counts with percentages. Group differences were examined using the chi‐square test or Fisher's exact test for categorical variables. For continuous variables, the Student's *t*‐test was used for comparisons between two groups, whereas one‐way analysis of variance (ANOVA) was used for comparisons involving three or more groups (e.g., SLD subtypes). Linkage‐to‐care and uptake of fibrosis testing were analysed according to diagnostic setting, SLD subtype and at‐risk status. The care cascade yield was calculated sequentially from the overall SLD population to the SLD with at‐risk population, subsequent clinical revisits and completion of fibrosis assessment. To identify independent predictors of linkage‐to‐care and fibrosis assessment, multivariable logistic regression analyses were performed. Both models were adjusted for age group, sex, BMI, geographic residence, SLD subtype, T2DM, hypertension, dyslipidemia, CKD and alcohol intake; the fibrosis assessment model additionally included care setting and at‐risk status. Results are expressed as odds ratios (OR) with 95% confidence intervals (CI). All statistical analyses were performed using SPSS (version 27; IBM Corp., Armonk, NY, USA). Statistical significance was defined as a two‐sided *p* value < 0.05.

## 3 Results

### 3.1 Self‐Reported Prevalence of SLD Was 23.7%

The self‐reported prevalence of SLD in Korea was 23.7%. Table 1 shows characteristics of the study population. The mean age was 47.8 ± 10.8 years, with 61.9% being men. The prevalence of comorbid conditions among SLD was 16.8% for T2DM, 36.3% for hypertension, 42.1% for dyslipidemia and 4.4% for chronic kidney disease, indicating a high burden of liver‐related risk and supporting the relevance of evaluating linkage‐to‐care and fibrosis risk assessment in this population. These comorbidity patterns were comparable to population‐level statistics in Korea [17, 18, 19]. Among SLD subtypes, MASLD accounted for 73.3% of cases, followed by MetALD (11.9%), Others (10.2%) and ALD (4.6%). Compared with the general Korean population, the study sample included a higher proportion of men (62.0% vs. 50.0%) and middle‐aged adults, consistent with known epidemiologic patterns of SLD (Table S2).

**TABLE 1:** Baseline characteristics.

Variables	Total (*n* = 1000)	MASLD (*n* = 733)	MetALD (*n* = 119)	ALD (*n* = 46)	Others (*n* = 102)	*p*
Men, *n* (%)	619 (61.9%)	451 (61.5%)	76 (63.9%)	28 (60.9%)	64 (62.7%)	0.962
Age, year	47.8 ± 10.8	47.9 ± 10.9	47.9 ± 10.0	45.4 ± 10.4	47.8 ± 11.1	0.489
BMI, kg/m^2^	26.0 ± 4.1	26.1 ± 4.3	25.9 ± 3.7	26.0 ± 3.8	25.0 ± 3.6	0.089
Urban residency, *n* (%)	617 (61.7%)	450 (61.4%)	75 (63.0%)	30 (65.2%)	62 (60.8%)	0.941
T2DM, *n* (%)	168 (16.8%)	125 (17.1%)	19 (16.0%)	10 (21.7%)	14 (13.7%)	0.663
HTN, *n* (%)	363 (36.3%)	257 (35.1%)	37 (31.1%)	19 (41.3%)	39 (38.2%)	0.562
Dyslipidemia, *n* (%)	421 (42.1%)	292 (39.8%)	53 (44.5%)	18 (39.1%)	40 (39.2%)	0.794
CKD, *n* (%)	44 (4.4%)	32 (4.4%)	7 (5.9%)	3 (6.5%)	2 (2.0%)	0.465
Initial diagnosis setting						
Health check‐up	799 (79.9%)	584 (79.7%)	103 (86.6%)	33 (71.7%)	79 (77.5%)	0.18
Primary clinic	96 (9.6%)	67 (9.1%)	9 (7.6%)	9 (19.6%)	11 (10.8%)	
Secondary/tertiary hospital	100 (10.0%)	77 (10.5%)	7 (5.9%)	4 (8.7%)	12 (11.8%)	
Other	5 (0.5)	5 (0.7)	0 (0)	0 (0)	0 (0)	
Linkage‐to‐care	577 (57.7)	407 (55.5)	71 (59.7)	30 (65.2)	69 (67.6)	0.08
At‐risk population	619 (61.9)	427 (58.3)	84 (70.6)	37 (80.4)	71 (69.6)	< 0.001

*Note:* *p* < 0.05 by Student *t*‐test or chi‐square test.

Abbreviations: ALD, alcoholic liver disease; BMI, body mass index; CKD, chronic kidney disease; MASLD, metabolic dysfunction‐associated steatotic liver disease; MetALD, metabolic dysfunction and alcohol‐related liver disease; SLD, steatotic liver disease; T2DM, type 2 diabetes mellitus.

### 3.2 Patient‐Reported Linkage‐to‐Care Was 57.7% Among Individuals With Self‐Reported SLD

**FIGURE 1:**
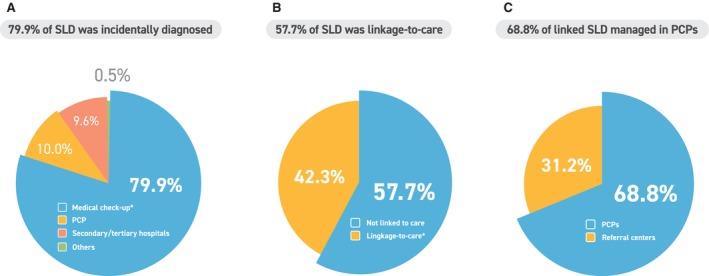
Patient‐reported diagnostic setting and linkage‐to‐care after a diagnosis of SLD. (A) Patient‐reported setting in which SLD was first identified among respondents with self‐reported physician‐diagnosed or imaging‐detected SLD (*n* = 1000), categorized as routine medical check‐ups, primary care clinics, secondary/tertiary hospitals or other routes. (B) Proportion of respondents who reported linkage‐to‐care after SLD identification versus those who reported no SLD‐related follow‐up. (C) Among respondents who reported linkage‐to‐care, the reported setting of follow‐up care was categorized as primary care clinics versus referral centres (secondary/tertiary hospitals). PCP, primary care physician; SLD, steatotic liver disease. *Detected incidentally during routine medical check‐ups.

Among individuals with self‐reported SLD, 79.9% (799/1000) reported that it was incidentally diagnosed during medical check‐ups conducted for reasons unrelated to liver disease (Figure 1A). In addition, 19.6% (196/1000) reported being diagnosed during liver‐related evaluations at primary clinics or referral hospitals, and 0.5% (5/1000) reported other routes of diagnosis. Among the 799 participants who reported an incidental diagnosis, 52.2% (417/799) reported no subsequent action, whereas 47.1% (376/799) reported visiting a primary care clinic or a secondary/tertiary hospital for further evaluation (other responses, *n* = 6, 0.7%). Overall, 57.7% (577/1000) achieved linkage‐to‐care, comprising 376 individuals who revisited for SLD evaluation after an incidental diagnosis, 196 diagnosed during liver‐related evaluations and five diagnosed through other routes. In contrast, 42.3% (423/1000) reported no SLD‐related follow‐up care or evaluation after diagnosis (i.e., no linkage‐to‐care) (Figure 1B). Among the 577 respondents reporting linkage‐to‐care, 68.8% (397/577) reported follow‐up in primary care clinics. The remaining 31.2% (180/577) reported receiving follow‐up care at referral centres (Figure 1C). On multivariable logistic regression, dyslipidemia (adjusted OR 1.69, 95% CI 1.27–2.25, *p* < 0.001), T2DM (adjusted OR 1.51, 95% CI 1.04–2.23, *p* = 0.033) and hypertension (adjusted OR 1.41, 95% CI 1.04–1.90, *p* = 0.027) were independently associated with linkage‐to‐care, after adjusting for age, sex, BMI, residence, SLD subtype and alcohol intake (Table S3). Age, sex, BMI, geographic residence, SLD subtype and alcohol intake were not independently associated with linkage‐to‐care. Among the 423 individuals who reported no follow‐up, the most commonly cited reason was the belief that SLD was not a serious condition (41.6%, 176/423), followed by the perception that it could be managed independently (23.9%, 101/423). Another frequently cited reason was not being advised by healthcare professionals to undergo further evaluation or testing (23.9%, 101/423) (Table S4).

### 3.3 Patient‐Reported Fibrosis Assessment Was 14.9% Among Those Who Reported Linkage‐to‐Care

Comparisons of clinical characteristics according to linkage‐to‐care status revealed no significant differences in sex, age, BMI, residential area, alcohol intake or SLD subtype between the two groups (Table 2). In contrast, metabolic comorbidities were significantly more prevalent among those who achieved linkage‐to‐care, specifically T2DM (20.5% vs. 11.8%, *p* < 0.001), hypertension (41.6% vs. 29.1%, *p* < 0.001), dyslipidemia (49.0% vs. 32.6%, *p* < 0.001) and chronic kidney disease (5.9% vs. 2.4%, *p* = 0.007). Linkage‐to‐care was achieved in 70.2% (118/168) of individuals with diabetes, 66.1% (240/363) with hypertension and 67.2% (283/421) with dyslipidemia. However, patient‐reported fibrosis assessment was 14.9% (86/577) among those who reported linkage‐to‐care. On multivariable analysis among patients linked to care, at‐risk status (adjusted OR 3.93, 95% CI 1.84–9.06, *p* < 0.001), SLD subtype ‘Others’ (adjusted OR 5.57, 95% CI 2.88–10.8, *p* < 0.001), management at a referral centre (adjusted OR 2.95, 95% CI 1.76–4.97, *p* < 0.001) and T2DM (adjusted OR 1.90, 95% CI 1.05–3.42, *p* = 0.032) were independently associated with fibrosis assessment uptake. Higher BMI was inversely associated with fibrosis assessment (adjusted OR 0.92 per kg/m^2^, 95% CI 0.85–0.99, *p* = 0.021) (Table S5).

**TABLE 2:** Clinical characteristics of individuals who achieved versus failed linkage to care.

Variables	Linkage to care		*p*
	No (*n* = 423)	Yes (*n* = 577)	
Men, *n* (%)	250 (59.1%)	369 (64.0%)	0.11
Age, year	47.0 ± 10.6	48.2 ± 10.8	0.07
BMI, kg/m^2^	26.0 ± 4.2	25.9 ± 4.0	0.57
Urban residency, *n* (%)	270 (63.8%)	347 (60.1%)	0.23
Alcohol intake, g/day	8.5 ± 13.5	8.6 ± 13.6	0.92
SLD type, *n* (%)			
MASLD	326 (77.1%)	407 (70.5%)	0.07
MetALD	48 (11.3%)	71 (12.3%)	
ALD	16 (3.8%)	30 (5.2%)	
Others	33 (7.8%)	69 (12.0%)	
T2DM, *n* (%)	50 (11.8%)	118 (20.5%)	< 0.001
HTN, *n* (%)	123 (29.1%)	240 (41.6%)	< 0.001
Dyslipidemia, *n* (%)	138 (32.6%)	283 (49.0%)	< 0.001
CKD, *n* (%)	10 (2.4%)	34 (5.9%)	0.007
Liver fibrosis assessment	0 (0.0%)	86 (14.9%)	< 0.001

*Note:* *p* < 0.05 by Student *t*‐test or chi‐square test.

Abbreviations: ALD, alcoholic liver disease; BMI, body mass index; CKD, chronic kidney disease; MASLD, metabolic dysfunction‐associated steatotic liver disease; MetALD, metabolic dysfunction and alcohol‐related liver disease; SLD, steatotic liver disease; T2DM, type 2 diabetes mellitus.

### 3.4 Follow‐Up Care Setting Among Those Reporting Linkage‐to‐Care: Primary Care Clinics vs. Referral Centres

**FIGURE 2:**
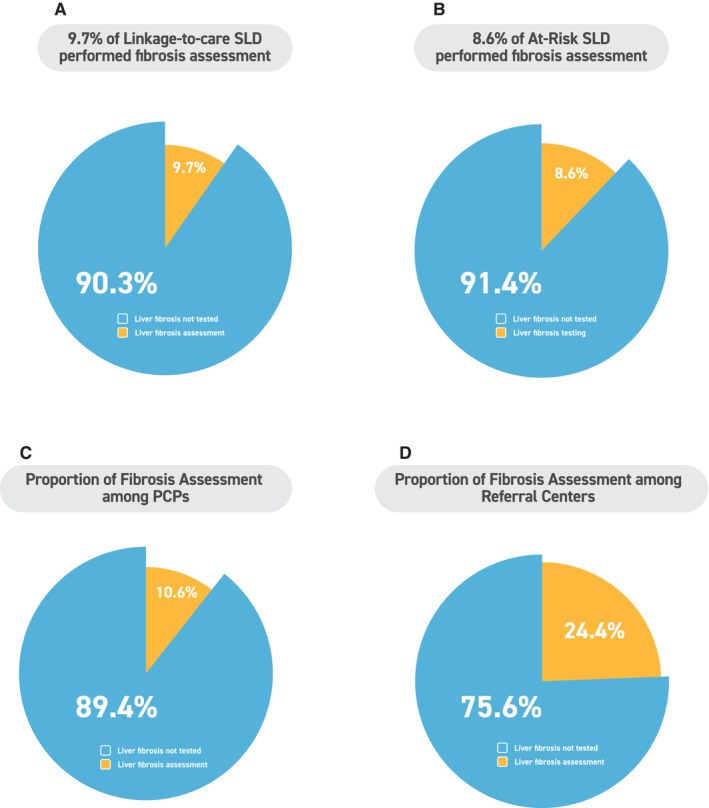
Patient‐reported uptake of fibrosis risk assessment after linkage‐to‐care and by care setting. (A) Proportion of respondents who reported undergoing fibrosis risk assessment among those who reported linkage‐to‐care. (B) Proportion of respondents who reported undergoing fibrosis risk assessment among the at‐risk subgroup. (C) Proportion of respondents who reported undergoing fibrosis risk assessment among those reporting follow‐up in primary care clinics. (D) Proportion of respondents who reported undergoing fibrosis risk assessment among those reporting follow‐up in referral centres. Fibrosis risk assessment was defined as patient‐reported receipt of imaging‐based elastography and/or non‐invasive blood‐based fibrosis tests, as assessed in the questionnaire. ‘Not tested’ indicates no patient‐reported fibrosis risk assessment. PCP, primary care physician.

**FIGURE 3:**
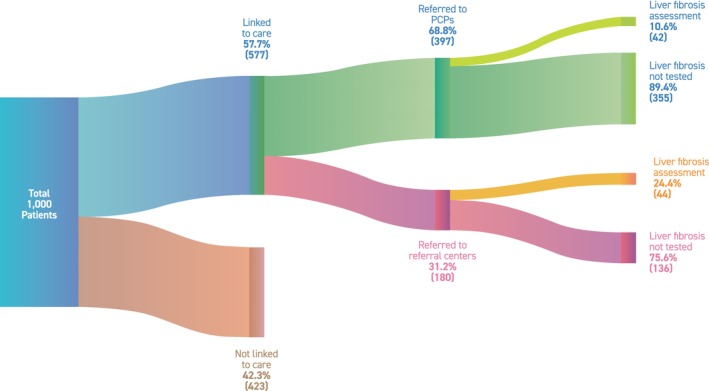
Care cascade of SLD: diagnosis, linkage‐to‐care and fibrosis risk assessment. Sankey diagram illustrating the patient‐reported care cascade among respondents with self‐reported SLD (*n* = 1000): initial identification of SLD, subsequent linkage‐to‐care versus no follow‐up, distribution of follow‐up setting (primary care clinics vs. referral centres) and the proportion reporting fibrosis risk assessment within each follow‐up setting. The width of each flow is proportional to the number of respondents represented at each step. PCP, primary care clinic/physician; SLD, steatotic liver disease.

Among the 397 individuals who reported follow‐up care in primary care, the patient‐reported rates of additional blood tests, imaging studies and fibrosis assessment after SLD diagnosis were 42.8%, 35.5% and 10.6%, respectively (Figures 2 and 3, Table S6). Among the 180 individuals who reported follow‐up care at referral centres, the patient‐reported rates of additional blood tests, imaging studies and fibrosis assessment were 41.1%, 50.6% and 24.4%, respectively (Figures 2 and 3, Table S6).

### 3.5 SLD With At‐Risk Population: Linkage‐to‐Care 65.6%, Fibrosis Assessment 12.1%

Of the 1000 individuals with SLD, 61.9% (619/1000) were classified as at‐risk, defined as having coexisting T2DM, a history of elevated liver enzymes on at least two occasions or obesity plus at least one additional cardiometabolic risk factor (Figure S2). Among this SLD with at‐risk population, 65.6% (406/619) achieved linkage‐to‐care. Compared with those SLD without at‐risk features (*n* = 381), individuals in the SLD with at‐risk group were more often men (68.3% vs. 51.4%) and had higher age and BMI (48.3 vs. 46.8 years; 27.0 vs. 24.2 kg/m^2^), as well as higher mean daily alcohol intake (9.5 vs. 6.9 g/day). Compared with those without at‐risk features, the at‐risk group reported higher linkage‐to‐care (65.6% vs. 44.9%) and higher patient‐reported fibrosis assessment (12.1% vs. 2.9%). Overall, 12.1% (75/619) of at‐risk respondents reported having undergone fibrosis assessment. Among at‐risk respondents who also reported linkage‐to‐care (*n* = 406), 18.5% (75/406) reported fibrosis assessment. These findings indicate that patient‐reported fibrosis assessment was uncommon even among respondents with higher‐risk features (Table S7).

### 3.6 Linkage‐to‐Care and Fibrosis Assessment Rate According to SLD Subtypes

Among the 1000 individuals diagnosed with SLD, the proportions classified as SLD with at‐risk were 58.3% (427/733) in MASLD, 70.6% (84/119) in MetALD, 80.4% (37/46) in ALD and 69.6% (71/102) in Others (*p* < 0.001, Table S8). Linkage‐to‐care rates did not differ significantly by subtype (55.5% [407/733] for MASLD, 59.7% [71/119] for MetALD, 65.2% [30/46] for ALD and 67.6% [69/102] for Others [*p* = 0.08, Table S8]). Among individuals who reported linkage‐to‐care, patient‐reported fibrosis assessment was 11.5% (47/407) in MASLD, 8.5% (6/71) in MetALD, 20.0% (6/30) in ALD and 39.1% (27/69) in Others (*p* < 0.001, Table S9).

## 4 Discussion

Among 12, 946 Korean adults aged 19 years or older, 23.7% reported having SLD, of whom 73.3% were classified as MASLD, 11.9% as MetALD and 4.6% as ALD. Among 1000 individuals with SLD, only 57.7% visited a healthcare facility after diagnosis and achieved linkage‐to‐care, while the remaining 42.3% failed to do so. Overall, 61.9% of those with SLD were classified as an SLD with at‐risk population. However, patient‐reported uptake of fibrosis risk assessment remained limited, including among at‐risk individuals for whom risk stratification is generally recommended. These findings suggest a potential unmet need for fibrosis risk assessment and/or patient awareness of such assessment in routine care.

Earlier studies have emphasized low awareness of MASLD/NAFLD and incomplete follow‐up in the general population [20, 21]. In contrast, in this survey, the self‐reported prevalence of physician‐diagnosed or imaging‐detected SLD was comparable to national estimates [22, 23], suggesting that recognition of SLD in the general population may be relatively common. While linkage‐to‐care was not universal, more than half of respondents reported some form of follow‐up within the healthcare system. Notably, patient‐reported fibrosis assessment was uncommon even after linkage‐to‐care. Reported uptake was lower in primary clinics (10.6%) than in referral centres (24.4%). Among the at‐risk population, 12.1% (75/619) reported having undergone fibrosis assessment, which was slightly lower than the corresponding rate among those linked to care overall (14.9%, 86/577). These patterns may reflect multiple factors, including limited access to elastography, variation in local practice and potential gaps in patient–clinician communication regarding fibrosis risk stratification. Importantly, guideline‐recommended first‐line risk stratification can include serum‐based indices (e.g., FIB‐4) calculated from routine laboratory tests, which patients may not recognize as a ‘fibrosis assessment’; therefore, patient‐reported rates in this study may underestimate the true frequency of fibrosis risk stratification in practice. Accordingly, the reported rates reflect patient‐reported awareness of fibrosis assessment rather than a direct measure of clinician‐initiated risk stratification. The true frequency of guideline‐concordant fibrosis evaluation in routine practice is therefore likely higher than observed in this survey. Taken together, these findings suggest that fibrosis risk stratification and subsequent assessment may not be consistently reaching—or being recognized by—individuals with higher‐risk features, particularly in primary care settings [24]. In an another data from primary care physicians and specialists, Lee et al. [25] reported that the proportion using VCTE for follow‐up of MASLD patients was only 16.3% in primary care and 38.9% even among tertiary care specialists, and that only 30%–40% of primary care physicians stated they would consider fibrosis assessment in high‐risk MASLD patients with obesity, dyslipidemia, diabetes or cardiovascular disease.

Similar losses along the care cascade have been reported in linkage pathway studies such as the iLFT program in the United Kingdom. Srivastava et al. [5] showed that approximately 35% of patients classified as high risk for liver fibrosis by non‐invasive testing were either not referred to or did not attend secondary care, and that linkage‐to‐care was also substantially incomplete among those at high risk according to enhanced liver fibrosis (ELF). Spann et al. [26] likewise reported that, in an electronic health record–based fibrosis‐4 index (FIB‐4) strategy for NAFLD/MASLD management, fewer than 3% of patients with abnormal FIB‐4 results were actually linked to elastography or specialist hepatology care. Collectively, these studies suggest that substantial attrition can occur along the care pathway even when structured algorithms are available, highlighting the need for practical, system‐level strategies to support risk stratification, appropriate referral and patient understanding of the evaluation process. In addition, systematic strategies are required to disseminate and embed established national and international guidelines, as well as non‐invasive fibrosis assessment algorithms, into routine primary care practice.

The present findings offer several insights into how post‐diagnostic care pathways for SLD might be strengthened. Linkage‐to‐care was driven predominantly by cardiometabolic comorbidity burden, suggesting that patients without established metabolic disease—who may nonetheless harbour significant fibrosis—are at particular risk of falling outside the care pathway; proactive risk‐stratification at the point of SLD diagnosis, rather than comorbidity‐triggered referral alone, may therefore be warranted. Regarding fibrosis assessment, the strong independent associations with referral‐centre care and at‐risk status reflect a structural disparity between primary and specialist settings, which may be partly addressed by embedding non‐invasive indices such as FIB‐4 into routine primary care workflows, thereby reducing reliance on specialist referral for initial risk stratification.

This study has several limitations. Assessment of liver fibrosis was based on self‐reported information. The specific testing modality was not captured, which prevented differentiation between serum‐based indices and imaging‐based elastography techniques. As this was a population‐based survey relying on participant recall, recall bias is inevitable. Furthermore, participants may not have been aware that a fibrosis assessment had been performed, potentially leading to underestimation of the true assessment rate. This includes uncertainty regarding the timing of SLD diagnosis and subsequent clinical evaluations. In addition, the lack of linkage to objective medical records may have resulted in some degree of misclassification. Because SLD was identified through self‐report, some respondents may not have accurately recalled or understood their diagnosis, and other liver conditions may have been inadvertently included. In addition, information on the timing of diagnosis and specific imaging modality was not captured, limiting assessment of the temporal relationship between diagnosis and subsequent care‐seeking behaviour. Although the condition‐list approach was designed to minimize framing bias and the observed prevalence was consistent with national estimates, the absence of objective diagnostic confirmation remains an inherent limitation of this study. The study sample was limited to 1000 individuals with SLD. However, this sample was derived from a broader screening of 12 946 adults from the general population, enhancing the robustness of the survey‐based estimates. Additionally, participants were recruited from an online research panel, which may introduce selection biases related to digital literacy and health awareness. Such biases would most likely lead to overestimation of linkage‐to‐care and fibrosis assessment rates. Therefore, the gaps identified in this study may be even more pronounced in the broader population. Finally, as the study was conducted in a Korean population, the generalizability of the findings to other populations and healthcare systems may be limited. Despite these limitations, the study has important strengths. It is a large, nationwide investigation conducted in a general population sample. The survey was developed through multidisciplinary collaboration. By focusing on patient‐reported experiences rather than physician‐reported practices, the study provides valuable insight into public awareness, real‐world diagnostic pathways and post‐diagnostic care trajectories of SLD. To our knowledge, this is the first studies to systematically examine linkage‐to‐care and guideline implementation for SLD at the population level. To our knowledge, this is the first study to systematically examine linkage‐to‐care and patient‐reported uptake of guideline‐recommended fibrosis risk assessment for SLD at the population level.

In conclusion, in this nationwide quota‐based survey, many individuals with self‐reported SLD reported no follow‐up after diagnosis, and patient‐reported uptake of fibrosis risk assessment was limited even among at‐risk individuals. These findings support the need for strategies to strengthen post‐diagnostic pathways, improve communication and patient understanding, and facilitate scalable non‐invasive fibrosis risk stratification in routine care, particularly in primary care settings.

## Author Contributions

**Joo Hyun Oh:** original writing. **Jun‐Hyuk Lee:** data acquisition. **Sang Bong Ahn:** supervise. **Eunjoo Kwon:** data analysis. **Eileen L. Yoon:** project administration. **Hyo Young Lee:** visualization. **Seon Cho:** methodology. **Dae Won Jun:** supervise, conceptualization.

## Funding

This research was supported by a grant of Research Program funded by the Korea National Institute of Health (grant number: 2025‐ER0902‐01), the National Research Foundation of Korea (NRF) grant funded by the Korea Government (MSIT) (RS‐2023‐00217123) and ‘Regional Innovation System & Education (RISE)’ through the Seoul RISE Center, funded by the Ministry of Education (MOE) and the Seoul Metropolitan Government (2025‐RISE‐01‐027‐01).

## Disclosure

Guarantor of the article: Dae Won Jun.

## Conflicts of Interest

The authors declare no conflicts of interest.

## Supporting information


**Figure S1:** Flow chart.
**Figure S2:** Care pathway among the at‐risk SLD subgroup: linkage‐to‐care and fibrosis risk assessment.
**Table S1:** Questionnaire.
**Table S2:** Baseline characteristics of participants with self‐reported steatotic liver disease (SLD) (*n* = 1000).
**Table S3:** Univariable and multivariable logistic regression analyses for predictors of linkage to care (*n* = 1000).
**Table S4:** Reasons for not achieving linkage to care after SLD diagnosis.
**Table S5:** Univariable and multivariable logistic regression analyses for predictors of fibrosis assessment uptake among patients linked to care (*n* = 577).
**Table S6:** Post‐diagnostic clinical evaluations after linkage to care according to care setting.
**Table S7:** Baseline characteristics and care patterns according to SLD risk status.
**Table S8:** Linkage to care, perceived barriers and liver fibrosis assessment according to SLD subtype.
**Table S9:** Liver fibrosis assessment according to SLD subtype.

## Data Availability

The data that support the findings of this study are available on request from the corresponding author. The data are not publicly available due to privacy or ethical restrictions.
