# Supplementary material for: The MASLD Journey in the General Population: Linkage‐to‐Care and Patient‐Reported Uptake of Fibrosis Risk Assessment
Source: Liver Int. 2026 Apr 20;46(5):e70658. doi: 10.1111/liv.70658 (PMC13093470; doi:10.1111/liv.70658)
Supplement: Supplementary file 1 — Figure S1: Flow chart. Figure S2: Care pathway among the at‐risk SLD subgroup: linkage‐to‐care and fibrosis risk assessment. Table S1: Questionnaire. Table S2: Baseline characteristics of participants with self‐reported steatotic liver disease (SLD) (n = 1000). Table S3: Univariable and multivariable logistic regression analyses for predictors of linkage to care (n = 1000). Table S4: Reasons for not achieving linkage to care after SLD diagnosis. Table S5: Univariable and multivariable logistic regression analyses for predictors of fibrosis assessment uptake among patients linked to care (n = 577). Table S6: Post‐diagnostic clinical evaluations after linkage to care according to care setting. Table S7: Baseline characteristics and care patterns according to SLD risk status. Table S8: Linkage to care, perceived barriers and liver fibrosis assessment according to SLD subtype. Table S9: Liver fibrosis assessment according to SLD subtype. [file LIV-46-0-s001.docx]

**The MASLD Journey in the General Population: Linkage-to-care and Patient-Reported Uptake of Fibrosis Risk Assessment**

Joo Hyun Oh, Jun-Hyuk Lee, Sang Bong Ahn, Eunjoo Kwon, Eileen L Yoon, Hyo Young Lee, Seon Cho, and Dae Won Jun

**
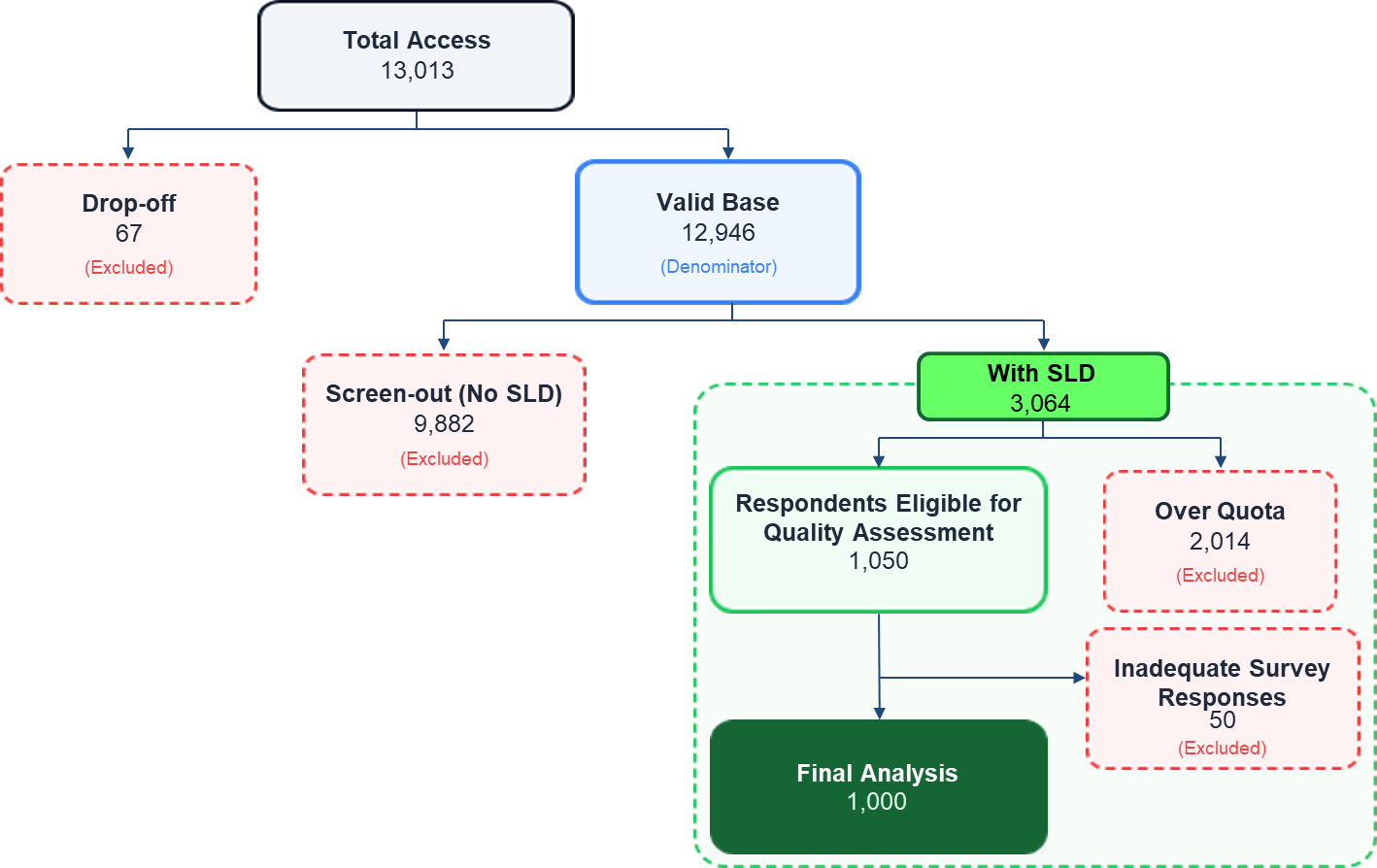
**

**Supplementary Figure 1.** Flow chart

Abbreviation: SLD, steatotic liver disease.

**
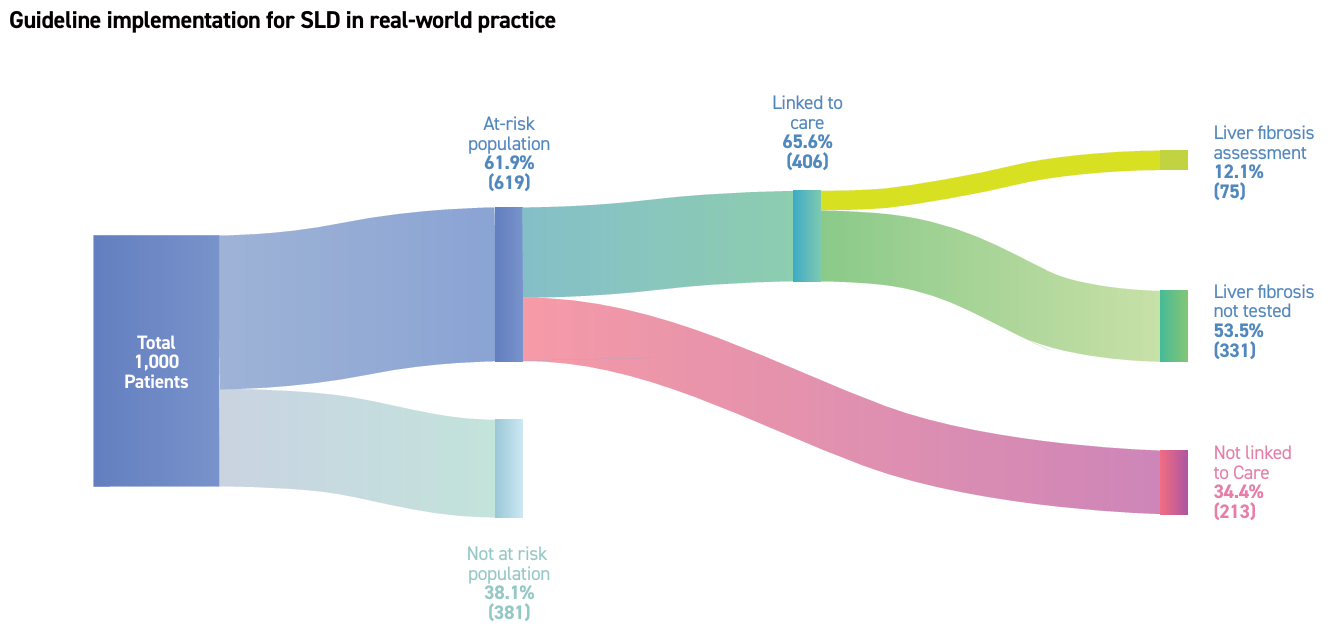
Supplementary Figure 2. Care pathway among the at-risk SLD subgroup: linkage-to-care and fibrosis risk assessment**

Sankey diagram showing the patient-reported care pathway for the at-risk subgroup among respondents with self-reported SLD (n=1,000): classification into at-risk versus not at-risk, linkage-to-care versus no follow-up within the at-risk subgroup, and patient-reported uptake of fibrosis risk assessment among those meeting at-risk criteria. The width of each flow is proportional to the number of respondents represented.

The at-risk subgroup was defined as meeting at least one of the following: type 2 diabetes mellitus; obesity (BMI ≥25 kg/m²) plus at least one additional cardiometabolic risk factor; or persistently elevated liver enzymes (ALT or AST >40 U/L on ≥2 occasions within the past 2 years).

Abbreivations: ALT, alanine aminotransferase; AST, aspartate aminotransferase; BMI, body mass index; SLD, steatotic liver disease.

**Supplementary Table 1.** Questionnaire

SQ1. What is your sex? [Select one]

1. Male 2) Female

SQ2. What is your age? Please enter your year of birth. [Answer by year of birth]
Year of birth ( ) = ______ years old (age in full years)
[Prog: Continue survey for respondents aged 20 years or older.]

SQ2_1. Age recode

1. 20–29 2) 30–39
2. 40–49 4) 50–59
3. 60–69 6) 70 or older

SQ3. What is your area of residence? [Show map]

SQ4. Please select all diseases/conditions you have ever been diagnosed with through medical care or a health check-up. [Multiple responses allowed]

1. Malignant tumor (e.g., stomach cancer, colorectal cancer, lung cancer, etc.)
2. Heart disease (e.g., myocardial infarction, angina, arrhythmia, etc.)
3. Kidney (renal) disease (e.g., dialysis, etc.)
4. Alcohol-related disease
5. Hepatitis B
6. Hepatitis C
7. Fatty liver disease
8. Thyroid disease
9. Colorectal adenoma (polyp)
10. Obstructive sleep apnea
11. None of the above
    [Prog: Continue survey for respondents who selected option 7.]

SQ5. Do you usually drink alcohol? [Select one]

1. Yes 2) No

[Prog: If SQ5 = 1]
SQ5-1. How much alcohol do you drink per week? [Select one]

1. Less than half a bottle
2. One bottle or less
3. Two bottles or less
4. Three bottles or less
5. More than three bottles
   [Prog: Continue survey for respondents selecting options 1–4.]

[Prog: Show the notice and questions on one page]
Consent Form for Collection and Use of Sensitive Information

Macromill Embrain Co., Ltd. collects sensitive information as follows for participation in this survey.

1. Purpose of collection and use: Collecting respondents’ opinions, verifying survey contents, etc.
2. Items of sensitive information collected: Information that may significantly infringe on privacy, including religion, race, ideology/beliefs, labor union/political party membership or withdrawal, political opinions, health, sex life, etc.
3. Retention and use period: One year after collection

Q0. You may refuse to consent to the collection of sensitive information; however, refusal may restrict your participation in the survey. [Select one]

1. I agree 2) I do not agree
   [Prog: Continue survey if option 1 is selected.]

Q1. Where were you first diagnosed with fatty liver or non-alcoholic fatty liver disease (NAFLD)? [Select one]

1. Health check-up
2. Local clinic
3. Hospital (general hospital or university hospital)
4. Other ( )

[Prog: If Q1 = 1]
Q2-1. After being diagnosed with “fatty liver” or “NAFLD” at a health check-up, what did you do? [Select one]

1. I was informed of fatty liver at the health check-up but took no particular action.
2. I was informed and revisited the same check-up center.
3. I was informed and visited a local clinic.
4. I was informed and visited a hospital or a higher-level/referral hospital.
5. Other ( )

[Prog: If Q1 = 2]
Q2-2. After being diagnosed with “fatty liver” or “NAFLD” at a local clinic, what did you do? [Select one]

1. I was informed at the local clinic but took no particular action.
2. I was informed and revisited the same local clinic.
3. I was informed and visited a hospital or a higher-level/referral hospital.
4. Other ( )

Q3. At the time you were diagnosed with fatty liver, did a healthcare professional explain that additional tests or management were needed? [Select one]

1. Yes, I was told I have fatty liver and received an explanation about additional tests or management.
2. I was told I have fatty liver, but I did not receive an explanation about additional tests or management.

[Prog: If Q3 = 1]
Q4. What explanation did you receive at the time of diagnosis? [Multiple responses allowed]

1. I was advised to undergo additional tests at the same clinic/hospital.
2. I was advised to improve lifestyle without any medication.
3. I was prescribed medication and advised to follow up.
4. I was advised to visit a higher-level hospital (or specialist).
5. I was advised to have a re-test after several months without any specific measures.
6. Other ( )

Q5. Did you visit a clinic or hospital on your own for additional tests or management of fatty liver? [Select one]

1. No. I did not make any additional visits or undergo further tests for fatty liver.
2. Yes. I made additional visits or underwent further tests.

[Prog: If Q5 = 1]
Q6. Why are you not receiving care at a clinic or hospital? [Multiple responses allowed]

1. I did not receive a clear explanation from medical staff about the need for management.
2. I received an explanation, but I did not think fatty liver is a serious disease.
3. I received an explanation, but I do not have enough time to visit the hospital.
4. Due to the financial burden of medical costs.
5. I believe I can manage it on my own based on internet searches, etc.

[Prog: If Q5 = 2]
Q7. If you received medical care at a hospital, what assessments or treatments did you receive? [Multiple responses allowed]

1. I underwent additional imaging tests such as ultrasound or abdominal CT.
2. I had additional blood tests.
3. I received diet/exercise education without specific medication or tests.
4. I was prescribed medication only, without specific tests.
5. I underwent a liver fibrosis test to determine how “stiff” or “hardened” the liver is.

Q8. Have you ever heard of, or do you know about, a “liver fibrosis test” that assesses how “stiff” or “hardened” the liver is? [Select one]

1. Yes 2) No 3) Not sure / I don’t remember

[Prog: If Q8 = 1]
Q9. Have you ever undergone a test related to liver fibrosis? [Select one]

1. Yes 2) No 3) I don’t remember

Q10. After your fatty liver diagnosis, did you make any lifestyle changes on your own? [Multiple responses allowed]

1. I changed my diet.
2. I started exercising.
3. I started taking supplements/health functional foods (e.g., Urusa, milk thistle, etc.).
4. No lifestyle changes.
5. Other ( )
   [Prog: If option 4 is selected, no other options may be selected.]

Q11. What difficulties do you have in improving and maintaining lifestyle changes? [Multiple responses allowed]

1. I know it is necessary, but I don’t have enough time.
2. I know it is necessary, but I lack willpower / it feels bothersome.
3. I know it is necessary, but I don’t know how to do it (lack of information).
4. I don’t really feel it is necessary.
5. Other ( )
   [Prog: If option 4 is selected, no other options may be selected.]

Q12. What treatments are you currently receiving to manage fatty liver? [Multiple responses allowed]

1. I am taking liver-protective agents or fatty liver-related medications prescribed by a hospital.
2. I am receiving prescription weight-loss drug treatment from a hospital.
3. I am taking supplements/health functional foods on my own.
4. I am only managing lifestyle.
5. I am doing nothing.
   [Prog: If option 4 or 5 is selected, no other options may be selected.]

Q13. If you currently have any other diseases, please select all that apply. [Multiple responses allowed]

1. Diabetes
2. Hypertension
3. Dyslipidemia (hyperlipidemia)
4. Stroke
5. Angina or heart disease
6. I am currently being treated for cancer or have been diagnosed with cancer in the past.
7. Other ( )
8. None

Q14. Have you ever been told that your liver enzymes (ALT, AST, or GPT, GOT) are elevated? [Select one]

1. Yes 2) No

[Prog: If Q14 = 1]
Q15. Have you been told two or more times that your liver enzymes are elevated? [Select one]

1. Yes 2) No

Q16. How often do you drink alcohol? [Select one]

1. Never
2. Once a month or less
3. 2–4 times a month
4. 2–3 times a week
5. 4 or more times a week
   [Prog: If SQ5 = 2, auto-punch Q16 option 1 and skip.]

[Prog: If Q16 = 2–5]
Q17. When you drink, how many drinks do you usually have per occasion? [Select one]

1. 1–2 drinks
2. 3–4 drinks
3. 5–6 drinks
4. 7–9 drinks
5. 10 or more drinks

[Prog: If Q16 = 2–5]
Q18. In the past 6 months, how often have you had 6 or more drinks on one occasion? [Select one]

1. Never
2. 1–2 times
3. Once a month
4. Once a week
5. Daily or almost daily

Q19. What is your height in cm? (To one decimal place, if you know it) [Numeric entry]
(125.0–225.0) cm

Q20. What is your weight in kg? (To one decimal place, if you know it) [Numeric entry]
(35.0–150.0) kg

**Supplementary Table 2.** Baseline characteristics of participants with self-reported steatotic liver disease (SLD) (n = 1,000)

| **Characteristic** | **Category** | **n (%)** | **Korean population (%)** | *P* |
| --- | --- | --- | --- | --- |
| Sex | Men | 620 (62.0%) | 50.0% | <0.001 |
|  | Women | 380 (38.0%) | 50.0% |  |
| Age group (years) | 20–29 | 37 (3.7%) | 11.9% | <0.001 |
|  | 30–39 | 213 (21.3%) | 13.5% |  |
|  | 40–49 | 303 (30.3%) | 14.9% |  |
|  | 50–59 | 290 (29.0%) | 16.8% |  |
|  | 60–69 | 137 (13.7%) | 15.2% |  |
|  | ≥70 | 20 (2.0%) | 13.2% |  |
| Region of residence | Seoul | 233 (23.3%) | 18.2% | 0.003 |
|  | Gyeonggi | 309 (30.9%) | 26.7% |  |
|  | Incheon | 75 (7.5%) | 5.9% |  |
|  | Busan | 59 (5.9%) | 6.4% |  |
|  | Daegu | 45 (4.5%) | 4.6% |  |
|  | Gwangju | 31 (3.1%) | 2.7% |  |
|  | Daejeon | 25 (2.5%) | 2.8% |  |
|  | Ulsan | 33 (3.3%) | 2.1% |  |
|  | Sejong | 3 (0.3%) | 0.8% |  |
|  | Gangwon | 21 (2.1%) | 3.0% |  |
|  | Chungcheongbuk-do | 36 (3.6%) | 3.1% |  |
|  | Chungcheongnam-do | 20 (2.0%) | 4.2% |  |
|  | Jeollabuk-do | 16 (1.6%) | 3.4% |  |
|  | Jeollanam-do | 27 (2.7%) | 3.5% |  |
|  | Gyeongsangbuk-do | 47 (4.7%) | 4.9% |  |
|  | Gyeongsangnam-do | 30 (3.0%) | 6.3% |  |
|  | Jeju | 9 (0.9%) | 1.3% |  |

**Supplementary Table 3.** Univariable and multivariable logistic regression analyses for predictors of linkage-to-care (n = 1,000)

|  | **Univariable** | | **Multivariable** | |
| --- | --- | --- | --- | --- |
| Characteristic | Unadjusted OR  (95% CI) | p-value | Adjusted OR  (95% CI) | p-value |
| Age group (ref: 40–59 years) |  |  |  |  |
| 20–39 years | 0.84 (0.62–1.13) | 0.3 | 1.06 (0.77–1.47) | 0.7 |
| ≥60 years | 1.02 (0.72–1.47) | >0.9 | 0.82 (0.56–1.20) | 0.3 |
| Sex, Female (ref: Male) | 0.81 (0.63–1.05) | 0.12 | 0.88 (0.66–1.16) | 0.4 |
| BMI (kg/m²) | 0.99 (0.96–1.02) | 0.6 | 0.97 (0.94–1.01) | 0.13 |
| Residence, Non-metropolitan (ref: Metropolitan) | 1.17 (0.90–1.52) | 0.2 | 1.21 (0.92–1.58) | 0.2 |
| SLD subtype (ref: MASLD) |  |  |  |  |
| MetALD | 1.18 (0.80–1.77) | 0.4 | 1.49 (0.83–2.70) | 0.2 |
| ALD | 1.50 (0.82–2.87) | 0.2 | 2.37 (0.95–6.48) | 0.075 |
| Others | 1.67 (1.09–2.63) | 0.022 | 1.53 (0.97–2.47) | 0.074 |
| Type 2 diabetes mellitus | 1.92 (1.35–2.76) | <0.001 | 1.51 (1.04–2.23) | 0.033 |
| Hypertension | 1.74 (1.33–2.27) | <0.001 | 1.41 (1.04–1.90) | 0.027 |
| Dyslipidemia | 1.99 (1.53–2.58) | <0.001 | 1.69 (1.27–2.25) | <0.001 |
| Chronic kidney disease | 2.59 (1.31–5.58) | 0.009 | 1.88 (0.92–4.14) | 0.10 |
| Alcohol intake (g/day) | 1.00 (0.99–1.01) | >0.9 | 0.99 (0.97–1.00) | 0.11 |

The multivariable model was adjusted for all variables listed in the table. Abbreviations: OR, odds ratio; CI, confidence interval; OR, odds ratio.

**Supplementary Table 4.** Reasons for not achieving linkage to care after SLD diagnosis

|  | No linkage to care  (n = 423) | Linkage to care  (n = 577) | *P* |
| --- | --- | --- | --- |
| Perceived SLD as non-serious despite explanation by healthcare providers | 176 (41.6) | 92 (15.9) | <0.001 |
| Believed self-management was sufficient | 101 (23.9) | 49 (8.5) | <0.001 |
| Lack of adequate explanation from healthcare providers | 101 (23.9) | 37 (6.4) | <0.001 |
| Lack of time for hospital visits | 37 (8.7) | 29 (5.0) | 0.019 |
| Financial burden | 12 (2.8) | 13 (2.3) | 0.56 |

Abbreviation: SLD, steatotic liver disease.

Data are presented as n (%)

^†^Referral centers include secondary and tertiary hospitals.

Primary care clinics include medical check-up centers and community-based primary clinics.

* p-values were calculated using the chi-square test.

**Supplementary Table 5.** Univariable and multivariable logistic regression analyses for predictors of fibrosis assessment uptake among patients linked to care (n = 577)

|  | **Univariable** | | **Multivariable** | |
| --- | --- | --- | --- | --- |
| Characteristic | Unadjusted OR  (95% CI) | p-value | Adjusted OR  (95% CI) | p-value |
| Age group (ref: 40–59 years) |  |  |  |  |
| 20–39 years | 0.84 (0.62–1.13) | 0.3 | 1.06 (0.77–1.47) | 0.7 |
| ≥60 years | 1.02 (0.72–1.47) | >0.9 | 0.82 (0.56–1.20) | 0.3 |
| Sex, Female (ref: Male) | 0.81 (0.63–1.05) | 0.12 | 0.88 (0.66–1.16) | 0.4 |
| BMI (kg/m²) | 0.99 (0.96–1.02) | 0.6 | 0.97 (0.94–1.01) | 0.13 |
| Residence, Non-metropolitan (ref: Metropolitan) | 1.17 (0.90–1.52) | 0.2 | 1.21 (0.92–1.58) | 0.2 |
| SLD subtype (ref: MASLD) |  |  |  |  |
| MetALD | 1.18 (0.80–1.77) | 0.4 | 1.49 (0.83–2.70) | 0.2 |
| ALD | 1.50 (0.82–2.87) | 0.2 | 2.37 (0.95–6.48) | 0.075 |
| Others | 1.67 (1.09–2.63) | 0.022 | 1.53 (0.97–2.47) | 0.074 |
| Type 2 diabetes mellitus | 1.92 (1.35–2.76) | <0.001 | 1.51 (1.04–2.23) | 0.033 |
| Hypertension | 1.74 (1.33–2.27) | <0.001 | 1.41 (1.04–1.90) | 0.027 |
| Dyslipidemia | 1.99 (1.53–2.58) | <0.001 | 1.69 (1.27–2.25) | <0.001 |
| Chronic kidney disease | 2.59 (1.31–5.58) | 0.009 | 1.88 (0.92–4.14) | 0.10 |
| Alcohol intake (g/day) | 1.00 (0.99–1.01) | >0.9 | 0.99 (0.97–1.00) | 0.11 |

The multivariable model was adjusted for all variables listed in the table. Abbreviations: OR, odds ratio; CI, confidence interval; OR, odds ratio.

**Supplementary Table 6.** Post-diagnostic clinical evaluations after linkage to care according to care setting

|  | Primary care clinic  (n = 397) | Referral centers  (n = 180) | *P* |
| --- | --- | --- | --- |
| Blood tests | 170 (42.8) | 74 (41.1) | 0.70 |
| Additional imaging studies (ultrasonography or computed tomography) | 141 (35.5) | 91 (50.6) | <0.001 |
| Lifestyle counseling only | 17 (4.3) | 6 (3.3) | 0.58 |
| Medication without further testing | 22 (5.5) | 10 (5.6) | 0.99 |
| Liver fibrosis assessment | 42 (10.6) | 44 (24.4) | <0.001 |

**Supplementary Table 7.** Baseline characteristics and care patterns according to SLD risk status

|  | No risk group  (n = 381) | At-risk group  (n = 619) | *P* |
| --- | --- | --- | --- |
| Men, n (%) | 196 (51.4) | 423 (68.3) | <0.001 |
| Age, year | 46.8 ± 11.4 | 48.3 ± 10.3 | 0.027 |
| Body mass index, kg/m^2^ | 24.2 ± 3.4 | 27.0 ± 4.1 | <0.001 |
| Urban residency, n (%) | 237 (62.2) | 380 (61.4) | 0.79 |
| Alcohol intake, g/day | 6.9 ± 11.7 | 9.5 ± 14.5 | 0.003 |
| SLD type, n (%) |  |  | <0.001 |
| MASLD | 306 (80.3) | 427 (69.0) |  |
| MetALD | 35 (9.2) | 84 (13.6) |  |
| ALD | 9 (2.4) | 37 (6.0) |  |
| Others | 31 (8.1) | 71 (11.5) |  |
| T2DM, n (%) | 0 (0) | 168 (27.1) | <0.001 |
| HTN, n (%) | 56 (14.7) | 307 (49.6) | <0.001 |
| Dyslipidemia, n (%) | 88 (23.1) | 333 (53.8) | <0.001 |
| CKD, n (%) | 10 (2.6) | 34 (5.5) | 0.032 |
| Initial diagnostic setting |  |  | 0.53 |
| Health check-up | 309 (81.1) | 492 (79.5) |  |
| Primary care clinic | 39 (10.2) | 60 (9.7) |  |
| Secondary/Tertiary hospitals | 33 (8.7) | 67 (10.8) |  |
| Linkage-to-care (%) | 171 (44.9) | 406 (65.6) | <0.001 |
| Post-diagnosis |  |  | 0.21 |
| Primary care clinic | 124 (72.5) | 273 (67.2) |  |
| Referral hospitals | 47 (27.5) | 133 (32.8) |  |
| Perceived SLD as non-serious despite explanation by healthcare providers | 135 (35.4) | 133 (21.5) | <0.001 |
| Believed self-management was sufficient | 74 (19.4) | 76 (12.3) | <0.001 |
| Lack of adequate explanation from healthcare providers | 75 (19.7) | 63 (10.2) | <0.001 |
| Lack of time for hospital visits | 22 (5.8) | 44 (7.1) | 0.40 |
| Financial burden | 10 (2.6) | 15 (2.4) | 0.84 |
| Liver fibrosis assessment | 11 (2.9) | 75 (12.1) | <0.001 |

Abbreviations: SLD, steatotic liver disease; BMI; body mass index; MASLD, metabolic dysfunction-associated steatotic liver disease; MetALD, metabolic dysfunction and alcohol-related liver disease; ALD, alcoholic liver disease; T2DM, Type 2 diabetes mellitus; CKD, chronic kidney disease.

**Supplementary Table 8.** Linkage to care, perceived barriers, and liver fibrosis assessment according to SLD subtype

| Variables | MASLD  (n = 733) | MetALD  (n = 119) | ALD  (n = 46) | Others  (n = 102) | *P* |
| --- | --- | --- | --- | --- | --- |
| Linkage to care | 407 (55.5) | 71 (59.7) | 30 (65.2) | 69 (67.6) | 0.08 |
| At-risk group | 427 (58.3) | 84 (70.6) | 37 (80.4) | 71 (69.6) | <0.001 |
| Perceived SLD as non-serious despite explanation by healthcare providers | 201 (27.4) | 30 (25.2) | 15 (32.6) | 22 (21.6) | 0.47 |
| Believed self-management was sufficient | 120 (16.4) | 18 (15.1) | 2 (4.3) | 10 (9.8) | 0.06 |
| Lack of adequate explanation from healthcare providers | 116 (15.8) | 7 (5.9) | 6 (13.0) | 9 (8.8) | 0.012 |
| Lack of time for hospital visits | 52 (7.1) | 6 (5.0) | 2 (4.3) | 6 (5.9) | 0.74 |
| Financial burden | 21 (2.9) | (0) | 1 (2.2) | 3 (2.9) | 0.31 |
| Liver fibrosis assessment | 47 (6.4) | 6 (5.0) | 6 (13.0) | 27 (26.5) | <0.001 |

Abbreviations: SLD, steatotic liver disease; MASLD, metabolic dysfunction-associated steatotic liver disease; MetALD, metabolic dysfunction and alcohol-related liver disease; ALD, alcoholic liver disease.

**Supplementary Table 9.** Liver fibrosis assessment according to SLD subtype

| Variables | MASLD | MetALD | ALD | Others | *P* |
| --- | --- | --- | --- | --- | --- |
| Liver fibrosis assessment among individuals linked to care (%) | 11.5 (47/407) | 8.5 (6/71) | 20.0 (6/30) | 39.1 (27/69) | <0.001 |
| Liver fibrosis assessment among at-risk group (%) | 9.8 (42/427) | 7.1 (6/84) | 16.2 (6/37) | 29.6 (21/71) | <0.001 |

Abbreviations: SLD, steatotic liver disease; MASLD, metabolic dysfunction-associated steatotic liver disease; MetALD, metabolic dysfunction and alcohol-related liver disease; ALD, alcoholic liver disease.
